# Case report: Total obstruction of the left main bronchus by a blood clot leads to pneumonectomy in a pregnant woman

**DOI:** 10.1097/MS9.0000000000001527

**Published:** 2023-11-20

**Authors:** Hicham Ziani, Amine Chibani, Zakariae Slaihi, Abdelkader Benhalima, Bouchra Armel, Hamza ElHamzaoui, Imane Elazzouzi, Amine Eddouali, Selma Ghattab, Manal Elarfaoui, Mustapha Alilou

**Affiliations:** a Department of Intensive Care Unit; b Unit of Critical Emergency Care, Hospital IBN SINA, Rabat, Morocc

**Keywords:** blood clot, bronchoscopy, massive hemoptysis, pneumonectomy, respiratory failure

## Abstract

**Introduction and importance:**

Airway obstruction resulting from blood clot formation is observed across various clinical scenarios and is often preceded by hemoptysis. This condition can significantly compromise respiratory function, potentially leading to life-threatening ventilatory distress.

**Case presentation:**

In this report, the authors present a case of acute airway obstruction associated with hemoptysis in an 18-week pregnant woman admitted to the emergency department for acute respiratory distress. Clinical and radiographic evidence strongly suggested the presence of an endobronchial blood clot causing focal airway obstruction. Diagnosis was confirmed through direct endoscopic evaluation.

**Clinical discussion:**

Initial attempts to remove the obstructing clot from the airway involved lavage, aspiration, and forceps extraction by using a bronchoscope. In cases in which these measures proved ineffective, other management strategies include rigid bronchoscopy, embolization, and surgical resection.

**Conclusion:**

Central airway obstruction is a critical condition caused by numerous factors such as tumours or blood clots. Treatment focuses on securing the airway, ensuring breathing, and using tools such as bronchoscopy for diagnosis and treatment. Surgery is considered a last resort when other methods are ineffective.

## Introduction

Highlights

- An 18-week pregnant woman presented with acute respiratory distress and moderate hemoptysis.
- Imaging revealed total obstruction of the left main bronchus by an endobronchial process.
- Diagnostic challenges arose in differentiating between possible tumour and blood clot as the cause.
- Therapeutic interventions included bronchial fibroscopy, haemostasis techniques, intubation, and emergency pneumonectomy with a biopsy.
- Maternal and foetal outcomes were positive, with the patient being discharged home.

Recurrent hemoptysis can form a large blood clot, leading to obstruction of the lobar or main stem bronchi.

Many etiologies are involved ranging from infectious causes (tuberculosis) to tumoral causes (Hodgkin lymphoma).

Hemoptysis associated with pregnancy further complicates the situation whether it be for the parturient or the foetus.

## Patient and observation

### Patient information

A 28-year-old patient, gravida 2 para 1, 18 weeks of gestation, with a history of hospitalization for pulmonary tuberculosis at the age of 7.

### Clinical findings

upon admission to the emergency department, the patient exhibited respiratory distress and moderate hemoptysis that had persisted for a week.

Initial clinical examination revealed a conscious patient with a Glasgow coma scale (GCS)=15, tachypneic at 35 cycles/min with a blood oxygen saturation at 79% on room air, auscultation revealed reduced vesicular murmur and vocal vibration throughout the left lung, tachycardia with a heart rate at 100 bpm, blood pressure (BP)=105 mmHg/64 mmHg with no signs of peripheral hypoperfusion.

Blood gas: pH=7.28, PCO2=43.7 mmHg, HCO3 =20.2 mmol/l, BE= −6.1 mmol/l, PO2=135 mmHg with Fio2=90%.

Laboratory results: Hb=9 g/dl, WBC=7400/µl, PLT=145.000 µ/l, Na+=138 mEq/l, K+=3.7 mEq/l, urea=0.16 g/l, creatinine=5.4 mg/l.

**Figure 1:**
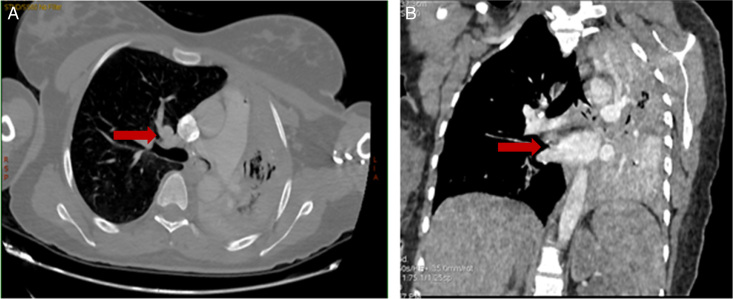
(A) Computed tomography (CT) chest: axial view showing left main stem bronchus obstruction (red arrow) with left sided atelectatic changes. (B) CT chest: coronal view showing left main stem bronchus occlusion (red arrow).

Imaging: A thoracic computed tomography (CT) angiography was performed, revealing complete atelectasis of the left lung with obstruction of the left main stem bronchus by a process, possibly a tumour or blood clot (Fig. 1).

### Therapeutic intervention

the patient was positioned in a semi-seated position and administered a high-concentration oxygen therapy at 10 l/min via a mask.

A bronchial fibroscopy was performed, revealing an impassable blood clot that bled upon contact, requiring the administration of adrenaline and ice-cold solutions to achieve haemostasis. A biopsy was then performed.

Following bronchoscopy, the patient began desaturating due to inhalation of the hemoptysis in the contralateral lung, with Spo2 at 60% and respiratory rate at 35 cycles/min under noninvasive ventilation with the following parameters Fio2=100%, inspiratory aid=18.

The patient was subsequently intubated, sedated, and ventilated.

Haemoglobin levels dropped to 6.5 g/dl leading to the introduction of vasoactive drugs (noradrenaline at 0,2 gamma/kg/min)

A foetal ultrasound was performed showing positive foetal cardiac activity.

**Figure 2:**
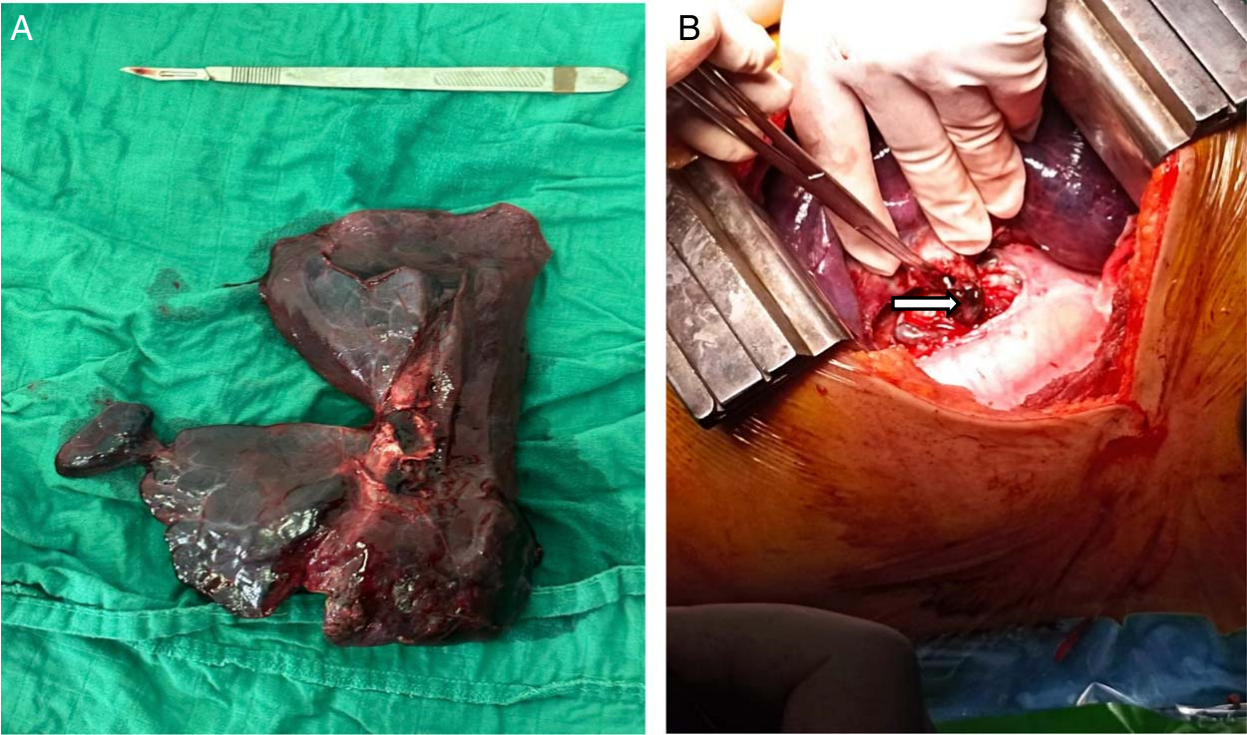
(A) Left radical pneumonectomy. (B) Surgical images of left pneumonectomy; blood clot obstructing the left main stem bronchus (white arrow).

Afterwards, thoracic surgery was requested, and a haemostasis pneumonectomy was performed by Professor A.Achir, head of the thoracic surgery department at Avicenne hospital. (Fig. 2).

### Follow-up and outcome of interventions

The thoracic surgery proceeded successfully, the patient was admitted to the intensive care unit and was extubated the following day, the foetal cardiac activity was positive. The patient was discharged home a week later with no further follow-up or surveillance done.

## Discussion

This work has been reported in line with the SCARE criteria^1^.

The search terms “Massive hemoptysis,” “Central airway obstruction,” “Endobronchial obstruction,” “Blood clot” and “Pregnant women” were instrumental in identifying relevant literature to understand the diagnostic and therapeutic challenges posed by recurrent hemoptysis, with the articles cited in this case report being sourced from the PubMed and ScienceDirect databases.

The literature review highlighted the significance of prompt interventions and the role of various medical and surgical approaches in managing cases of massive hemoptysis.

Massive hemoptysis is a high mortality acute disease, posing diagnostic and therapeutic challenges.

The definition of massive hemoptysis varies widely in the literature, from blood loss volumes of 200–1000 ml per day^2^. However, hemoptysis should be evaluated not in terms of the volume of bleeding but from the standpoint of the threat it poses to the patient’s life^3^.

Among cases of massive hemoptysis, the inciting cause of death is not the haemorrhagic shock but rather asphyxiation and heart failure from the inability to oxygenate or ventilate due to airway obstruction caused by the haemorrhage^4^.

The underlying aetiology of hemoptysis may involve the airway, the pulmonary parenchyma, or the pulmonary veins themselves. The most common overall cause of hemoptysis is airway disease with bronchiectasis, chronic bronchitis, and lung cancer being frequent culprits, although the specific aetiology varies among populations^5,6^.

In a study done by Wipa Reechaipichitkul and Sirikan Latong the most common cause of massive hemoptysis was bronchiectasis accounting for 33.7% of cases, followed by active tuberculosis accounting for 20.8% of cases and finally neoplasia accounting for 10.9% of cases, with the latter including both bronchogenic carcinoma and metastasis to the lung^7^. While the most common fungal infection that causes massive hemoptysis is aspergilloma with rates ranging from 50 to 90%^8^.

As in our case, ~20% of cases of hemoptysis^9,10^, remain without an aetiological diagnosis even after bronchoscopy and chest CT, leading to the classification of the case as idiopathic or cryptogenic hemoptysis^5,6,9^.

Massive and untreated hemoptysis carries a high mortality. The emergency first aid treatment is resuscitation and supportive measures. Patients should be treated in an intensive care unit, monitoring vital signs and oxygen saturation. The ideal time for bronchoscopy is still contentious. The consensus is an indication of emergency bronchial therapy following bronchoscopic suction and exploration of the lungs in patients with worsening hypoxia; whereas delayed bronchoscopy is preferred in stable patients on conservative management, especially when bleeding lesions are indicated by chest CT and angiography.

Bronchial measures and interventional angiography have become increasingly more accepted approaches to patient care^2,11,12^. Surgical resection is usually performed only after these measures fail in patients who are considered good candidates for surgery, even though surgery seems to be a more promising strategy than conservative treatment^13^.

The mortality rate after emergency lung resection is still high^9,10,14,15^. Aspiration pneumonia can compromise the pulmonary reserve, and while extensive lung resection is associated with a high mortality rate^15^, it is difficult to limit the extent of lung resection to minimize sacrifice of functional lung tissue.

Death from massive hemoptysis is typically the result of acute hypoxemic respiratory failure due to obstructive clot formation in the central airways. Therefore, priorities include not only haemostatic control but also evacuation of blood products from the conducting airways to maintain sufficient gas exchange.

Several reports in literature described endobronchial obstruction following episodes of hemoptysis^16^. The first confirmed case of endobronchial obstruction from blood clot was reported by Wilson in 1929^17^. The case involved a 23-year-old woman with tuberculosis and atelectasis who developed acute respiratory distress 3 days after onset of recurrent hemoptysis. Physical examination and chest radiograph findings were consistent with right middle and lower lobe collapse. She spontaneously expectorated 6 days later three extensive bronchial casts composed of clot, with rapid improvement of pulmonary symptoms.

In our case, the patient, at 18 weeks of a pregnancy, presented with respiratory distress and hemoptysis of thick blood clots. She underwent bronchoscopy to locate a source, but none was clearly found; a biopsy was performed. Immediately after the procedure, the patient developed massive hemoptysis with hypoxia. She was intubated and stabilized with spontaneous cessation of her haemorrhage. Immediate angiography revealed no active extravasation. Later, she again developed spontaneous massive hemoptysis. Manual ventilation via the endotracheal tube became impossible. Immediate bronchoscopy identified a blood clot extending from the main carina to the left main stem bronchus. The clot was not removed, and due to the persistence of massive hemoptysis, an emergency haemostasis pneumonectomy was performed successfully, with no aetiologic diagnosis established and the case being classified as an idiopathic hemoptysis.

It is also important to underline the weaknesses of this case report, these include a lack of detailed follow-up information and a limited pathological findings from the biopsy. We believe that emphasizing these weaknesses will provide a more nuanced understanding of the case’s limitations, highlighting areas for further research and improvement in the management of similar clinical scenarios.

### Similar cases overview

Here is a table summarizing similar cases found throughout the literature (Table 1).

**Table 1:** Case comparison

Refernces	Year	Age and sex	Patient presentation	Diagnostic findings	Therapeutic interventions	Outcome
Arney *et al.* ^16^	1999	53. Male	Peak inspiratory pressures to 90 cm H2O, difficulty in ventilation in an intubated patient	A large blood clot at the carina, extending into and partially obstructing both main stem bronchi	Removal of the clots using endobronchial Forceps	No further obstructive events occurred
Arney *et al.* ^16^	1999	54. Female	Massive hemoptysis following thoracentesis for community acquired pneumonia	Complete opacification of right hemithorax in chest radiography Revealed to be hemothorax. Endobrachial blood clot revealed through flexible bronchoscopy	Endobronchial lavage and forceps extraction were unsuccessful in complete removal. A repeat bronchoscopic evaluation 3 days later showed no evidence of the clot.	Patient remained hemodynamically stable with peak inspiratory pressures ranging from 35 to 38 cm H2O
Arney *et al.* ^16^	1999	33. Male	Massive bleeding in an intubated patient following third lower lobe transbronchial biopsy. Minimal pink frothy secretions emanating from endotracheal tube + diffuse wheezing	Bronchoscopy through endotracheal tube revealed clot obscuring the distal orifice of the endotracheal tube	Removal of the endotracheal tube and re-intubation showing that the clot extended down both mainstream bronchi and the entire endobronchial tree	Patient wheezing resolved after removal of tube and blood clot cast. Arterial blood gases normalized. Patient extubated within 24H without difficulty
Wilson^17^	1997	23. Female	Positive tubercle bacilii in sputum Respiratory distress 3 days after onset of recurrent hemoptysis	Chest X-ray showed atelectasis of the right middle and lower lobe	None	Rapid disappearance of the atelectasis after patient spontaneously expectorated bronchial cast made by blood clot 6 days later

## Conclusion

Central airway obstruction is a critical emergency linked to various conditions like tumours, oedema, mucus, or blood clots. Hemoptysis often signals its presence, but some cases remain asymptomatic, while others necessitate mechanical ventilation. Immediate actions involve securing the airway and ensuring proper oxygenation. Flexible fiberoptic bronchoscopy, accessible at the bedside, is pivotal for both diagnosis and treatment. CT pulmonary angiography serves as an alternative when bronchoscopy is unavailable or inconclusive while surgical resection remains as a last resort when other methods prove ineffective.

## Ethical approval

This is a case report talking about a rare situation but derived from “standard” clinical practice so an ethics board approval was not required.

## Informed consent

Informed consent was approved by the patient being fully aware of the stakes of this case report and wanting willingly to be a part of it.

## Source of funding

None.

## Author contribution

Writing the paper: Z.H. Data collection: C.A., S.Z., A.E. Proofreading: S.G., E.I. Internal review: E.H., M.A. Contributors: A.B., B.A., M.E.

## Conflicts of interest disclosure

The authors have no conflicts of interest to declare.

## Guarantor

Dr Ziani Hicham is the guarantor of this case report.
